# Cytokine Profiles in Human Metapneumovirus Infected Children: Identification of Genes Involved in the Antiviral Response and Pathogenesis

**DOI:** 10.1371/journal.pone.0155484

**Published:** 2016-05-12

**Authors:** Jostein Malmo, Nina Moe, Sidsel Krokstad, Liv Ryan, Simon Loevenich, Ingvild B. Johnsen, Terje Espevik, Svein Arne Nordbø, Henrik Døllner, Marit W. Anthonsen

**Affiliations:** 1 Childhood Airway Infections Research Group, Department of Laboratory Medicine, Children’s and Women’s Health, Faculty of Medicine, Norwegian University of Science and Technology, 7491 Trondheim, Norway; 2 Childhood Airway Infections Research Group, Department of Medical Microbiology, St. Olav’s Hospital, Trondheim University Hospital, 7491 Trondheim, Norway; 3 Centre of Molecular Inflammation Research, Department of Cancer Research and Molecular Medicine, Faculty of Medicine, Norwegian University of Science and Technology, 7489 Trondheim, Norway; 4 Childhood Airway Infections Research Group, Children’s Clinic, St. Olav’s Hospital, Trondheim University Hospital, 7491 Trondheim, Norway; University of Iowa, UNITED STATES

## Abstract

Human metapneumovirus (hMPV) causes severe airway infection in children that may be caused by an unfavorable immune response. The nature of the innate immune response to hMPV in naturally occurring infections in children is largely undescribed, and it is unknown if inflammasome activation is implicated in disease pathogenesis. We examined nasopharynx aspirates and blood samples from hMPV-infected children without detectable co-infections. The expression of inflammatory and antiviral genes were measured in nasal airway secretions by relative mRNA quantification while blood plasma proteins were determined by a multiplex immunoassay. Several genes were significantly up-regulated at mRNA and protein level in the hMPV infected children. Most apparent was the expression of the chemokine IP-10, the pro-inflammatory cytokine IL-18 in addition to the interferon inducible gene ISG54. Interestingly, children experiencing more severe disease, as indicated by a severity index, had significantly more often up-regulation of the inflammasome-associated genes IL-1β and NLRP3. Overall, our data point to cytokines, particularly inflammasome-associated, that might be important in hMPV mediated lung disease and the antiviral response in children with severe infection. Our study is the first to demonstrate that inflammasome components are associated with increased illness severity in hMPV-infected children.

## Introduction

The negative sense single strand RNA virus human metapneumovirus (hMPV) has since its discovery in 2001 emerged as a commonly detected respiratory tract pathogen involved in airway disease mainly affecting young children [1,2]. Numerous studies have shown that 5–10% of respiratory tract infections in children are related to hMPV [3]. Currently, four different hMPV genotypes have been identified: A1, A2, B1, and B2, where A2 has been divided into subgroups A2a and A2b [4]. Similar as for the closely related respiratory syncytial virus (RSV) [5] it has been proposed that the different hMPV genotypes induce disease of variable severity, but this is a matter of debate [6–8]. Despite normally causing mild disease in the upper airways, hMPV infections in the lower respiratory tract resulting in bronchiolitis or pneumonia are occasionally observed [8,9].

The molecular mechanisms involved in hMPV mediated lung pathogenesis and interaction with the host immune system remain largely unknown. Further, most of the limited number of studies available have been performed in cellular or animal models and studies based on human material are lacking. The severe cases of hMPV infection could be caused by an expression pattern of interferons (IFNs) and pro-inflammatory cytokines promoting pathological lung tissue damage, as reported for RSV [10]. Moreover, other possible mediators of severe hMPV infection are inflammasomes [11,12]. In addition to providing an important defense mechanism against infections, activation of the inflammasome has been shown to result in unfavorable pathogenic events e.g. after infection with influenza virus in animal lung [13]. The inflammasome is a multicomponent protein complex involved in the innate immune response and is activated by intracellular pathogens such as viruses [11]. The active inflammasome is able to cleave pro-IL-1β and pro-IL-18 into their active forms resulting in secretion of these pro-inflammatory cytokines and activation of specific receptors on cells. Several NLR proteins have been identified including NLRP3, which is involved in the antiviral response to respiratory tract viruses such as influenza- and RS-virus [14,15].

Herein, we evaluated the airway antiviral response in hMPV infected children. To this end, we determined the gene expression of type I, II and III IFNs, IFN-β, IFN-γ and IL-28 in nasopharyngeal aspirates (NPAs) and protein levels in blood plasma from hMPV positive children hospitalized with respiratory tract infection. Importantly, the NPAs were confirmed to be hMPV single-positive without viral co-infections. Further, we determined the expression of genes involved in the inflammasome-mediated antiviral response: IκBα, IL-1β, IL-18 and NLRP3. We also evaluated the presence of the chemokines IP-10, Gro-α, and Gro-β, the pro-inflammatory cytokines TNF-α and IL-6 in addition to the type I/III IFN-induced ISG54. The results from the gene expression analysis were compared to clinical data condensed into a severity index. This comparison indicated a correlation between the severity of disease and increased expression of inflammasome-associated genes. This study is the first to concomitantly determine mRNA expression and protein levels of inflammatory genes and inflammasome components in children hospitalized with acute hMPV infection.

## Materials and Methods

### Collection of nasopharyngeal aspirates and virus detection

During the time period January, 2007 to August, 2014, 2656 children with respiratory tract infection (RTI) were admitted to Children’s Clinic, St. Olav’s Hospital, Trondheim University Hospital. An NPA was sampled from all these children and a control group consisting of children admitted for elective day-surgery. All NPAs (including those from the control group) were collected in virus transport media (Hank’s balanced salt solution with 1% bovine serum albumin and fungizone) without antibiotics, and were cultured for viruses in vitro in appropriate cell lines. These cultures were evaluated for signs of CPE possibly caused by pathogens not detected by the PCR-assays. The samples included in this study were confirmed to be CPE-negative.

The NPAs were tested using PCR for hMPV (A1, A2a/b, B1, B2), adenovirus, human bocavirus-1, coronavirus (OC43, 229E and NL63), enterovirus, influenza A and B virus, parainfluenza virus type 1–4, parechovirus, RSV, rhinovirus, *Bordetella pertussis*, *Chlamydophila pneumoniae* and *Mycoplasma pneumoniae*. Nucleic acid was extracted using NucliSENS easyMAG according to the manufacturer’s protocol (bioMerieux). All PCRs were in-house real-time assays based on TaqMan probes [16]. For hMPV detection, primers targeting the N-gene allowing detection of all four hMPV genotypes were used as described elsewhere [17]. Genotyping was performed by sequencing an amplified F-gene PCR product [18] using a 3130 Genetic Analyzer (Applied Biosystems) and comparing sequenced data with the nucleotide BLAST database (www.ncbi.nlm.nih.gov/BLAST/).

### Patients and controls

A total of 162 children tested positive for hMPV (6.1%) in NPA. For the purpose of the present study, 30 children satisfying all of these criteria were recruited: an NPA was sampled less than one week after onset of symptoms and within one day after admittance, either hMPV genotypes A2 or B2 were detected in NPA, no viral co-infections detected in NPA, and serum levels of CRP <100 mg/L. Clinical data were recorded prospectively or retracted from medical records. Upper respiratory tract infection (URTI) was diagnosed when rhinitis, pharyngitis, tonsillitis and/or otitis media (serous, simplex or purulent) was found without signs of lower respiratory tract infection (LRTI). LRTI was diagnosed in children with signs and symptoms of respiratory difficulty such as tachypnea, retractions and nasal flaring, signs of lower airway obstruction (wheezing, prolonged expiration, rhonchi), focal findings (crepitations) and/or a positive chest x-ray (infiltrates, atelectasis, air trapping). LRTI was divided in bronchiolitis (age <2 years, tachypnea, retractions, wheezing, crepitations), obstructive bronchitis (age >2 years, signs of lower airway obstruction), asthma exacerbation with signs of lower airway obstruction, and pneumonia (cough, tachypnea, localized crepitations, x-ray infiltrates). A clinical severity score previously used to determine hMPV/RSV disease severity in children [7,8] was calculated for each infected child as the sum of these variables 1) length of hospital stay ≥5 days (1 point), 2) oxygen demand (1 point), 3) ventilatory support by continuous positive airway pressure ventilation (1 point), and 4) ventilatory support by endotracheal intubation and overpressure ventilation (2 points).

Ten children admitted for elective surgery with no symptoms of RTI in the last two weeks were randomly selected and included as controls. The clinical and virological findings are summarized in Table 1, and a detailed overview for the individual patients is presented in the S1 Table.

**Table 1:** Clinical and virological characteristics of the patients and controls.

Characteristics	Patients, n = 30	Controls, n = 10	P-value
**Age (months) (median, range)**	17.5 (0.7–91.9)	34.5 (3.9–57.4)	0.12^a^
**Male gender (number)**	18	8	0.25^b^
**Diagnoses (number)**			
**Upper respiratory tract infection**	2		
**Lower respiratory tract infection**	8		
**Upper and lower respiratory tract infection**	20		
**Classification of lower respiratory infection**			
**1. Bronchiolitis**	13		
**2. Pneumonia**	6		
**3. Obstructive bronchiolitis**	1		
**4. Asthma exacerbation**	6		
**5. Unspecified LRTI**	2		
**Length of hospital stay (days) (average, s.d.)**	3.6 (3.3)		
**Highest temp (°C) (median, range)**	38.8 (37.0–40.6)		
**Clinical severity score (median, range)**	0 (0–4)		
**Max C-reactive protein (mg/L) (median, range)**	30 (<5–95)		
**hMPV genotype (number)**			
**1. A2a**	7		
**2. A2b**	8		
**3. B2**	15		
**Ct-value (average, s.d.)**			
**1. A2a**	22.1 (7.1)		
**2. A2b**	24.3 (2.8)		
**3. B2**	26.8 (5.2)		

Abbreviations: Ct, cycle threshold; hMPV, human metapneumovirus; LRTI, lower respiratory tract infection; S.d., standard deviation. Statistical significance is indicated with the P-value from the a) Mann-Whitney or b) Pearson’s chi square test.

### mRNA quantification

cDNA was synthesized from RNA isolated from the collected NPAs using the qScript kit according to the manufacturer’s protocol (Quanta). Quantitative PCR (qPCR) was performed using Perfecta SYBR Green reaction mix (Quanta) and a StepOnePlus instrument (Life Technologies) with the temperature profile 95°C for 20 s, 40 cycles at 95°C for 3s and 60°C for 30s. Fold-change in IFN-β, IFN-γ, IL-28, IκBα, IL-1β, IL-18, NLRP3, IP-10, TNF-α, IL-6 and ISG54 gene expression relative to the control with highest levels of target gene mRNA was calculated using the ddC_t_-method with GAPDH as housekeeping gene. Data was plotted as box-plots where the box represents 50% of the values, the horizontal line indicates the median, whiskers show the maximum and minimum values, and the closed circles indicate outliers. Primer sequences used in this study are shown in the S2 Table.

### Protein quantification

Blood samples were collected in Vacuette EDTA tubes (Greiner Bio-One) and the fractions separated according to the manufacturer’s protocol. The concentrations of proteins in the plasma fraction were measured using a Bio-Plex multiplex system (Bio-Rad) and cytokine assays for IFN-β, IFN-γ, IL-28A, IL-1β, IL-18, IP-10, TNF-α, IL-6, Gro-α and Gro-β (Bio-Rad).

### Data analysis

Categorical variables were analyzed with the Pearson chi-square test or the 2-tailed Fisher’s exact test. Continuous and nearly normal distributed variables were analyzed with the Student’s t-test or ANOVA-test, and non-parametric variables were compared by Mann-Whitney U tests. A two-sided P-value less than 0.05 was considered statistically significant. Normally distributed and continuous variables are presented as average±standard deviation and non-normally distributed variables as median (range). Analyses were performed using the Statistical Package of Social Science (SPSS, version 19.0) and SigmaPlot (version 12.0).

### Ethics

The study was approved by the Regional Committee for Medical and Health Research Ethics (REK, Mid-Norway). Caregivers to all patients and controls received written information about the study. Written consent was obtained at the hospital from the caregivers of all controls and the majority of the patients included in the cohort. Due to practical challenges by enrolling patients 24 hours a day, 7 days a week, some patients were enrolled after discharge from the hospital. Their caregivers received written information after the hospital stay and children were included if the caregivers did not resist enrollment by taking contact to the hospital within 4 weeks.

## Results

This study was based on a cohort involving 2656 children hospitalized with respiratory tract infection (RTI) where 162 tested positive for hMPV. From the hMPV positive children we selected samples without detectable co-infections (n = 30) as described in the methods section. Further, children admitted for elective surgery without recent symptoms of RTI were randomly selected and included as controls (n = 10). Table 1 shows that the majority of the children were diagnosed with lower respiratory tract infection (bronchiolitis or pneumonia), and the disease severity ranged from 0–4. One half of the patients had more severe disease with a score higher than zero (S1 Table). During the period of this study, A2 (43% of the samples) and B2 (42%) were the dominant genotypes found in the cohort and therefore our focus. The length of hospitalization was average 4.1±4.2 days for A2 and 3.1±2.0 days for B2 infected children (P>0.05, Student’s t-test). The maximum C-reactive protein (CRP) values in the patients ranged from approximately 5 to 100 mg/L with the majority below 50 mg/L, indicating that severe bacterial infections were unlikely.

**Fig 1:**
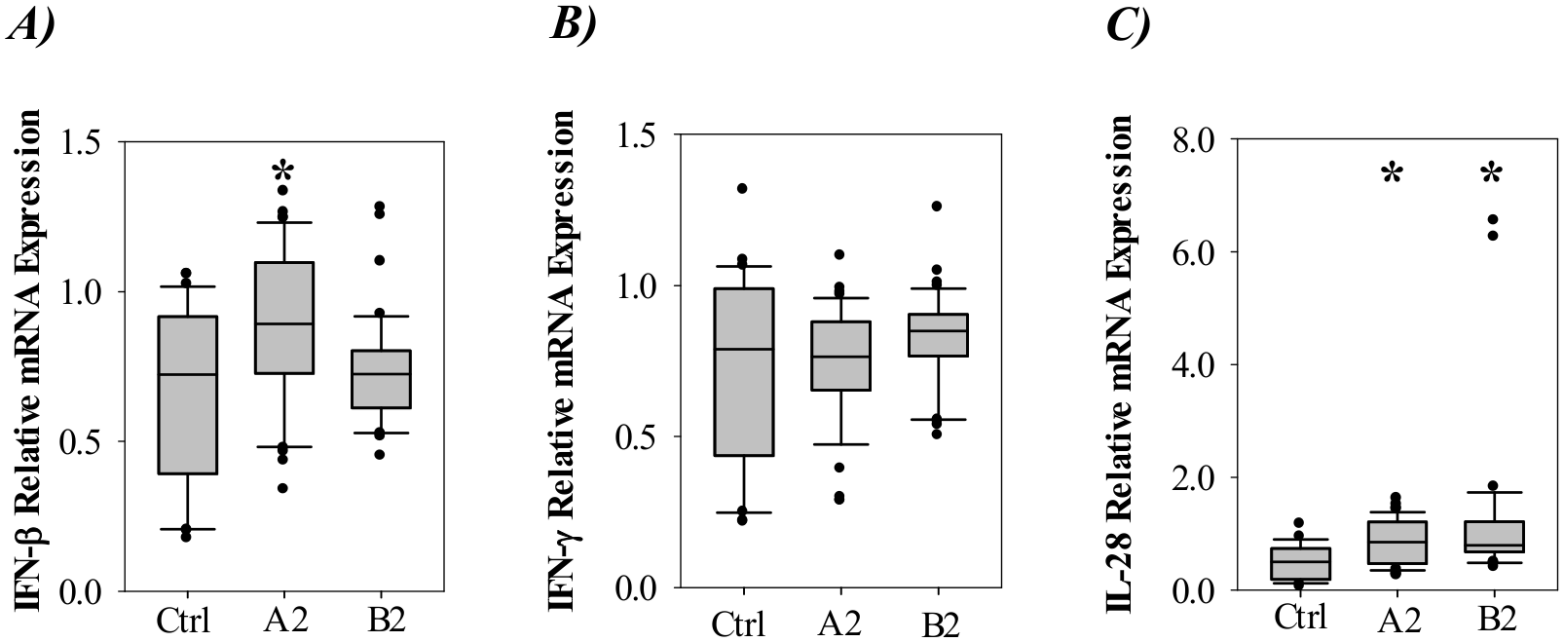
Expression of type I, II and III interferons in nasopharyngeal aspirates from controls or children infected with hMPV genotype A2 or B2. Relative levels of A) IFN-β (type I) B) IFN-γ (type II) and C) IL-28 (type III) mRNA expression. Data was plotted as box-plots where the box represents 50% of the values, the horizontal line indicates the median, whiskers show the maximum and minimum values, and the closed circles indicate outliers. Asterisk indicates statistical significant difference (P<0.05, ANOVA test).

To determine the presence of antiviral cytokines in children infected with hMPV and controls, we initially investigated the expression of type I, II and III IFNs. Fig 1A shows that only A2 infected children had slightly elevated mRNA levels of the type I IFN-β compared to the controls. As shown in Fig 1B, the mRNA levels of the type II IFN-γ was not elevated in any of the patient groups relative to the controls. In correspondence with this, IFN-γ protein was not detected in the blood samples from any of the investigated children (data not shown). Finally we evaluated the mRNA expression of the type III IFN IL-28. Fig 1C shows that both the A2 and B2 positive patients had significantly increased levels of IL-28 expression.

**Fig 2:**
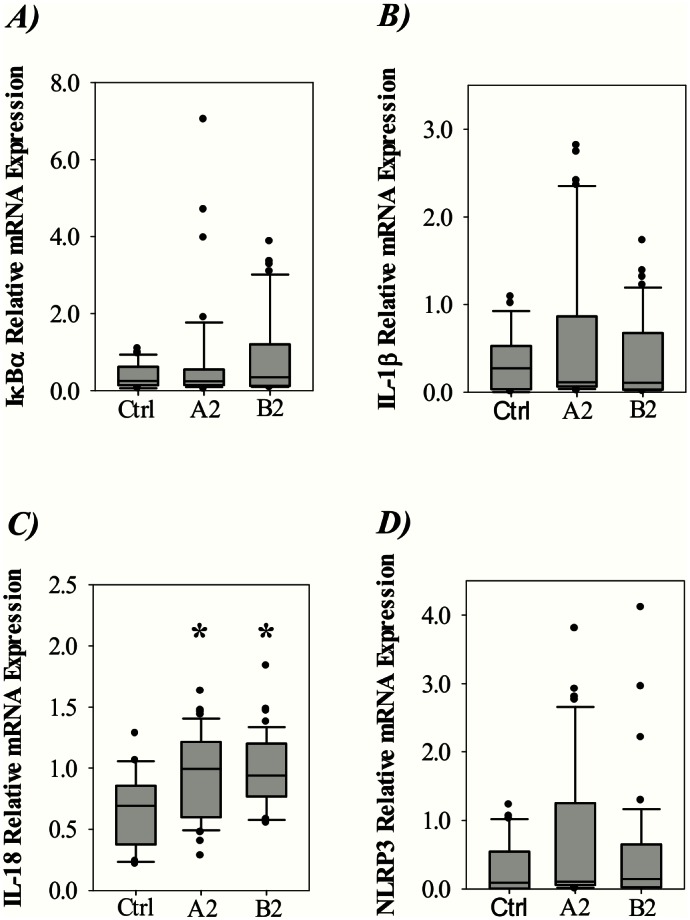
Expression of inflammasome-associated genes in nasopharyngeal aspirates from controls or children infected with hMPV genotype A2 or B2. Relative levels of A) IκBα, B) IL-1β, C) IL-18 and D) NLRP3 mRNA expression. Data was plotted as box-plots where the box represents 50% of the values, the horizontal line indicates the median, whiskers show the maximum and minimum values, and the closed circles indicate outliers. Asterisk indicates statistical significant difference (P<0.05, ANOVA test).

Next, we wanted to investigate the possible involvement of NF-κB and inflammasome-associated genes in response to hMPV infection. Fig 2 shows the mRNA expression of A) IκBα, a repressor gene induced by NF-κB activation [19], B) IL-1β, C) IL-18 and D) NLRP3 in hMPV infected children and controls. The expression of IL-18 was significantly increased for both A2 and B2 infected children. However, for the other genes investigated their expression was not significantly increased compared to the controls.

**Fig 3:**
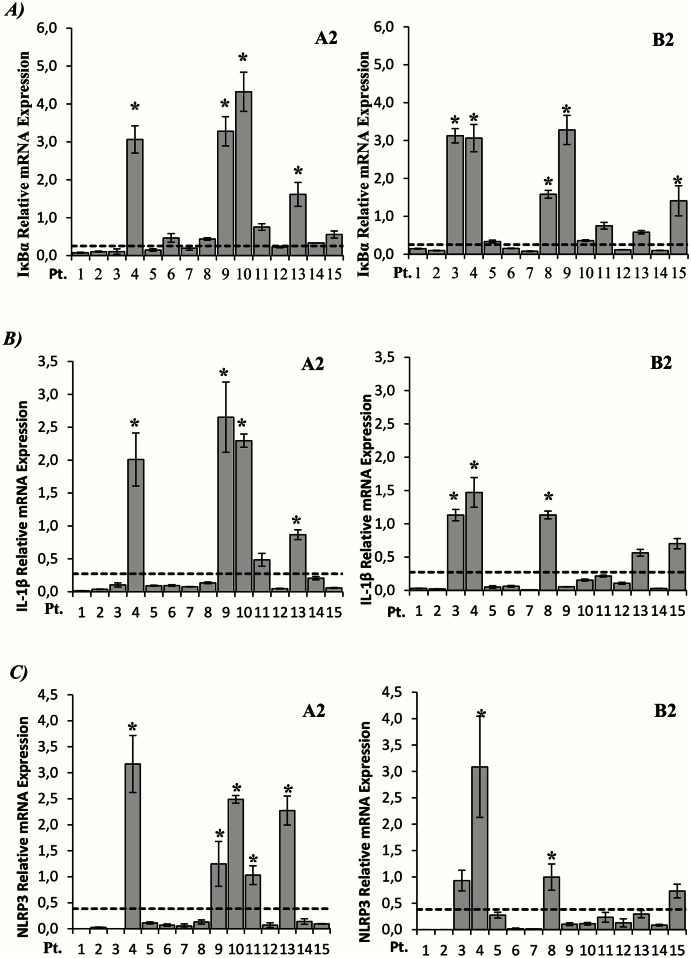
Individual variation in the expression of inflammasome-associated genes in hMPV infected children. Relative levels of A) IκBα, B) IL-1β and C) NLRP3 mRNA expression relative to the controls. The median level of expression for the controls is indicated with the dotted line. Asterisk indicates statistical significant difference (P<0.05, ANOVA test).

It should be noted that the standard deviations for the IκBα, IL-1β and NLRP3 expression were high. This was caused by a considerable fraction of the hospitalized children having undetectable expression while others had high mRNA levels relative to control subjects. In contrast, children from the control group exhibited similar levels of IκBα, IL-1β and NLRP3 mRNA levels. To illustrate this individual variation, differences in gene expression for individual A2 and B2 positive patients are outlined in Fig 3. From this figure, it can be seen that A) IκBα, B) IL-1β and C) NLRP3 were significantly upregulated in several patients. Interestingly, most of the patients with upregulation of IκBα, IL-1β and NLRP3 had increased expression of all three genes.

**Fig 4:**
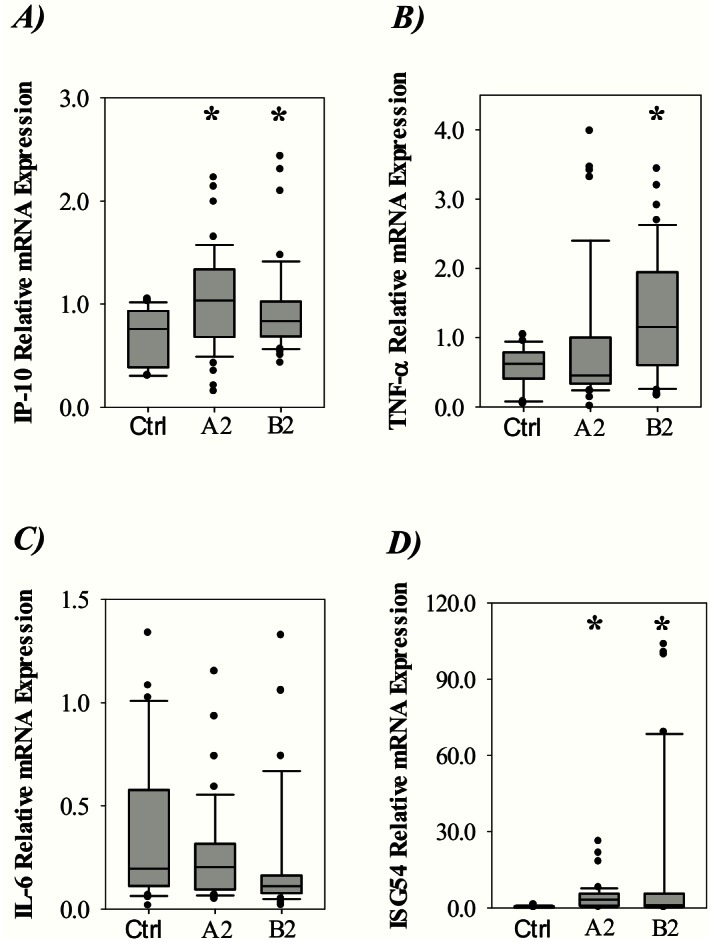
Expression of pro-inflammatory genes in nasopharyngeal aspirates from controls or children infected with hMPV genotype A2 or B2. Relative levels of A) IP-10, B) TNF-α, C) IL-6 and D) ISG54 mRNA expression. Data was plotted as box-plots where the box represents 50% of the values, the horizontal line indicates the median, whiskers show the maximum and minimum values, and the closed circles indicate outliers. Asterisk indicates statistical significant difference (P<0.05, ANOVA test).

To evaluate the involvement of other relevant immunomodulator genes, we measured the mRNA expression of IP-10, TNF-α, IL-6 and ISG54. As shown in Fig 4A, the expression of IP-10 was increased for both the A2 and B2 infected patients. The TNF-α expression (Fig 4B) was significantly higher in patients infected with B2 compared to the controls. Further, the expression of IL-6 (Fig 4C) was not increased at mRNA level in any of the groups with hMPV infected children. Finally, the expression of ISG54 (Fig 4D) was markedly increased for both A2 and B2 infected patients.

As mentioned earlier, the expression of inflammatory genes can possibly promote pathogenic events during RTI. Consequently, we wanted to investigate if the gene expression profiles differed in children with a severity index of zero compared to those with more serious disease and a higher severity score. As summarized in Table 2, the mRNA expression of IL-1β and NLRP3 were more frequently upregulated in the children with a severity score of 1–4.

**Table 2:** Fraction with significantly up-regulated genes at mRNA level in children with severity score 1–4 compared to children with severity score 0.

	Gene	
Severity score	IL-1β	NLRP3
**>0**	6/15 (40)	6/15 (40)
**0**	1/15 (7)	1/15 (7)
**Sum**	7/30 (23)	7/30 (23)
**P-value**	0.043	0.043

The percentage with up-regulated genes is given in the brackets. Statistical significance is indicated with the P-value from a Fisher’s exact test comparing the two groups.

**Fig 5:**
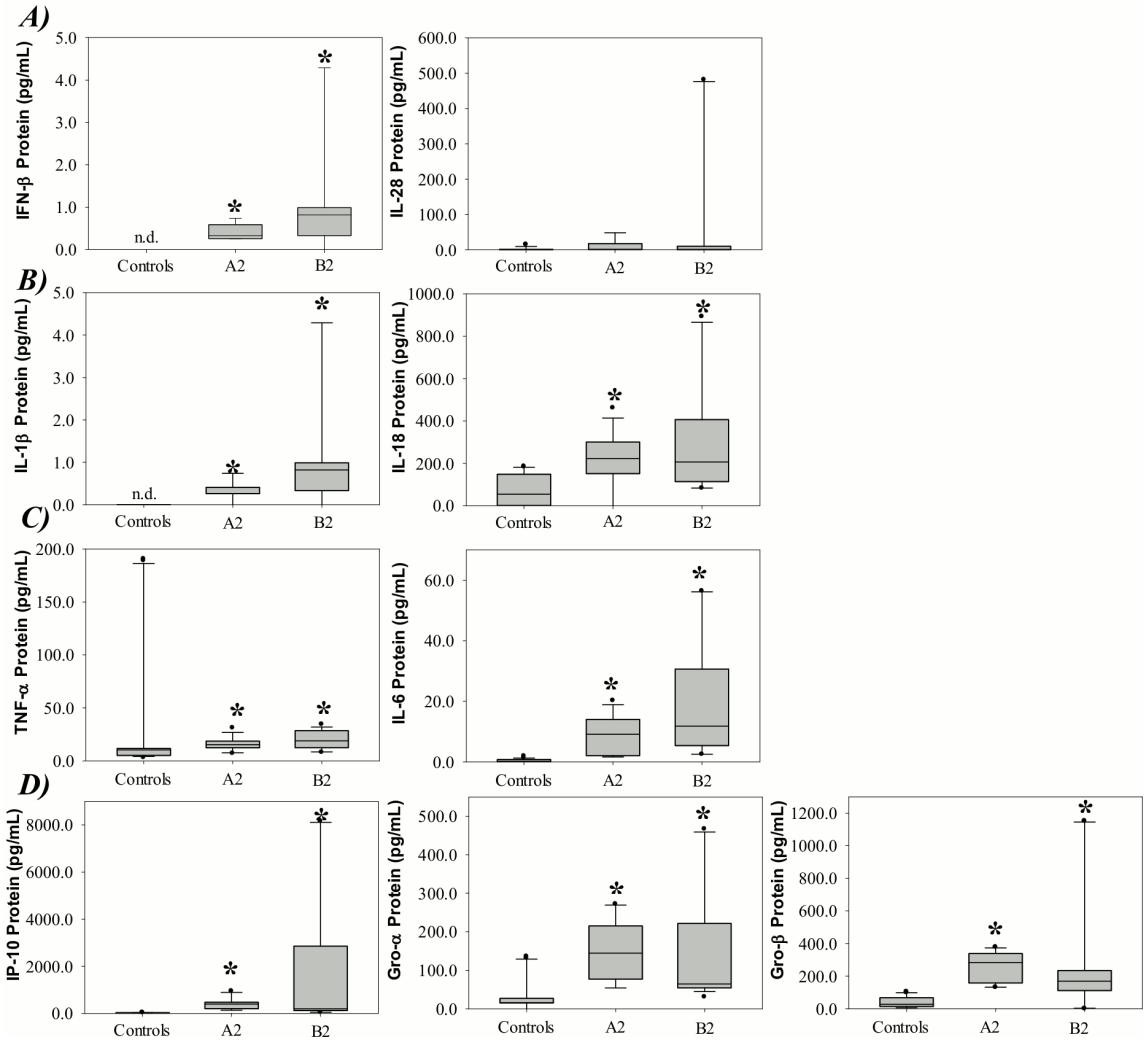
Levels of protein in blood serum collected from controls or children infected with hMPV genotype A2 or B2. Concentration of the A) Type I, II and III interferons IFN-β, IL-28 and IL-1β, respectively, B) Inflammasome-associated genes IL-1β and IL-18, C) Chemokines IP-10, Gro-α and Gro-β, and D) Pro-inflammatory genes TNF-α and IL-6. Data was plotted as box-plots where the box represents 50% of the values, the horizontal line indicates the median, whiskers show the maximum and minimum values, and the closed circles indicate outliers. Asterisk indicates statistical significant difference (P<0.05, ANOVA test). N.d. not detected.

Finally, the concentration of several secreted cytokines in blood plasma was measured for a random subset of the patients (n = 10) and the controls (n = 10) as shown in Fig 5. Herein, we also included the chemokines Gro-α and Gro-β to evaluate implications of neutrophil recruitment to the lungs. Fig 5 shows that all of the cytokines included in the analysis, except for IL-28, were detected at significantly higher concentrations in patients compared to the controls. Further, as mentioned earlier, IFN-γ was not detected above the threshold value in the controls or patient samples.

## Discussion

This study aimed to identify inflammatory genes associated with hMPV mediated disease. We performed an analysis of NPAs and blood samples collected from children hospitalized with RTI. Herein, we identify specific cytokines, such as IP-10, IL-18, TNF-α, and ISG54, that are elevated in hMPV mediated lung disease. Of noteworthy interest is the up-regulation of IL-1β and NLRP3 in samples from children with the most severe respiratory tract infection. This suggests that the inflammasome is involved in the innate immune response to hMPV in these patients, either as a protective mechanism or as a response to lung damage.

Type I and III IFNs are responsible for the induction of an antiviral state in cells immediately after infection [20]. While type I IFNs are produced by many different cell types, type III IFNs seems to be mostly produced by plasmacytoid dendritic and epithelial cells where the epithelial cells are the main targets for activity [21]. Due to their powerful and potentially harmful effects, the production of these cytokines is tightly regulated [22]. Our results showed that the type I IFN-β mRNA in NPA was marginally up-regulated for the A2 infected patients, but the protein concentrations in plasma were markedly increased for both A2 and B2 positive patients. This inconsistency could be explained by the protein being produced by tissue resident cells in e.g. the lung or lymph nodes. Another explanation could be a lag in the induction of protein synthesis in the epithelial cells after the elevation of mRNA transcription, since it is assumed that cytokines are able to enter the bloodstream from lung tissue during inflammation. For type III IFN IL-28 we observed increased mRNA expression both for A2 and B2 infected children. However, IL-28 protein was not significantly elevated in blood plasma. IL-28 is prominently expressed in lung epithelial cells, and suggested to prevent viral invasion through skin and mucosal surfaces [23]. Hence, the lack of increased IL-28 protein in blood could, similar as suggested for IFN-β, be explained by a lag in the induction of protein synthesis.

The expression of IL-28 has been shown to be triggered by viral infection, and it displays immunological properties similar to the type I IFN-α/β [23]. Recently, it has been shown that hMPV induces expression of IL-28 in mice and the cytokine was suggested to have an important protective role [24]. For RSV, which is phylogenetically and clinically closely related to hMPV, an in vitro study has shown that IL-28, in contrast to IFN-α/β, is induced during infection of primary respiratory tract cells [25]. Further, a study on infants hospitalized with RSV infection and bronchiolitis showed induced expression of IL-28 in the airways [26]. Overall, our results adds to existing data suggesting that IFN-β and IL-28 are induced to some extent during hMPV infection.

Chemoattractants are induced during acute inflammation events to recruit effector cells of the immune system. In our study, elevated mRNA expression of the chemoattractant IP-10 was observed for A2 and B2 infected patients. Further, elevated levels of IP-10 protein, and also of the chemokines Gro-α and Gro-β were found in blood plasma. IP-10 has previously been shown to be significantly elevated seven days post infection with hMPV A2 in mice [27]. The expression of IP-10 may be induced by IFN-γ [28], IFN-α/β [29] or TNF-α [30] secreted by different immune-cells. In our study, despite elevated IP-10, we did not measure any increased expression of the type II interferon IFN-γ at mRNA or plasma protein levels. The lack of IFN-γ detection in NPAs from hMPV infected children has also recently been reported elsewhere [31]. Our data suggests that IFN-β and TNF-α, which was significantly detectable in plasma, might be responsible for induction of IP-10. In addition, IFN-γ-independent induction of IP-10 has been demonstrated in human peripheral blood mononuclear cells [32]. In this setting the type III interferon IL-29, which is closely related to IL-28 that was shown to be upregulated herein, was correlated to IP-10 induction. Of note, when infecting human epithelial cells with hMPV in vitro, we observed that IL-28 mRNA was induced and that IL-29 showed a similar trend (data not shown), as also suggested elsewhere [24]. Hence, IL-28/29 could mediate IFN-γ independent IP-10 induction in hMPV infection. IFN-γ-independent IP-10 expression has also previously been reported in BAL samples from patients during respiratory tract virus infection [33].

We found that mRNA expression of the pro-inflammatory cytokine TNF-α was elevated only in the B2-infected children. However, the level of TNF-α protein in plasma was significantly increased both for A2 and B2 infected patients. An explanation for this could be different expression kinetics for mRNA and protein. TNF-α induces NF-κB [34] and, among other functions, it is involved in the recruitment of neutrophilic cells to the lungs [35]. TNF-α production has previously been shown to be at high levels in nasopharyngeal secretions from RS- or influenza-virus infected children [36]. When comparing the patients with a severity index of 0 and those with an index of 1–4, TNF-α had significantly higher levels of expression (P<0.001, Student’s t-test). This could indicate that the presence of TNF-α in the respiratory tract directly or indirectly affects the severity of disease in hMPV infected children. It is worth mentioning that the hMPV C_t_ value was similar for the patients with severity index 0 and higher (S1 Table), suggesting similar extent of viral replication in these patients.

We did not detect any increase in mRNA levels of the pro-inflammatory cytokine IL-6. On the other hand, the levels of IL-6 in blood plasma were significantly increased for the hMPV infected patients. Similar as for IFN-β, this observation could be explained by IL-6 protein being produced by tissue-resident cells or, alternatively, downregulation of gene expression after a peaked response.

A previous study comparing the expression of several inflammatory cytokines in hMPV, RSV and influenza virus, detected elevated levels of TNF-α, IL-6 and IL-1β protein in nasal washes from infants with RTI [9]. Together with our data showing increased detection of these proteins in blood from patients with RTI compared to the controls, this could suggest that at least a part of the protein detected in blood might have been secreted from cells in the respiratory tract.

The antiviral cytokine ISG54 was expressed at the highest relative levels of any of the genes included in this study. The mRNA expression was up-regulated for both hMPV A2- and B2-positive NPA samples. ISG54 is known to be induced by viruses downstream of pattern recognition receptors and IFNs [37]. The expression of ISGs is initiated by type I and type III interferons such as IFN-β and IL-28. We did indeed find that these IFNs were up-regulated at mRNA and/or protein level in hMPV infected children. The functional effects and clinical involvement of ISG54 are largely unknown. Nevertheless, ISG54 has been shown to inhibit viral replication in vitro and in vivo [38]. Interestingly, mice lacking ISG54 have recently been shown to be subject to high mortality when infected with the mouse respiratory tract pathogen Sendai virus—a negative sense, single-stranded RNA paramyxovirus [39]. This suggests that ISG54 has a protective function and limits pathogenesis in mice. However, in our study, we were not able to identify any differences with respect to length of hospitalization or severity index when comparing the groups of patients expressing ISG54 or not (P>0.05, Student’s t-test), suggesting a general protective function of this cytokine in children.

Our study reveal that most of the hMPV-infected children exhibited low levels of mRNA coding for inflammasome-associated proteins, except for IL-18 which was induced in both A2 and B2 infected patients, both at mRNA and protein levels. Interestingly, some patients showed distinct expression profiles with elevated levels of several genes involved in the inflammasome mediated immune-response. More specific, approximately 1/4 of the patients showed increased expression of IκBα, IL-1β and NLRP3. As mentioned earlier, the repressor gene IκBα expression is induced by NF-κB activation [19]. NF-κB induces mRNA expression of the potent pro-inflammatory cytokines pro-IL-1β and pro-IL-18 [40]. Further, increased level of NLRP3 mRNA expression is the initial essential step in activating the NLRP3-inflammasome. Taken together, the increased levels of NLRP3 mRNA in this group of children along with enhanced IL-1β and IL-18 mRNA expression and protein secretion could suggest that the NLRP3-inflammasome is activated to produce IL-1β and IL-18 in the respiratory tract of these patients. Previously, it has been shown that influenza virus is able to activate the NLRP3-inflammasome in mice [14]. This was found to be critical for survival and reduction of viral titers in mice lung. Further, RSV was recently shown to activate the NLRP3-inflammasome in vitro using primary human lung epithelial cells [15]. Our results show that the hMPV infected children with a severity score exceeding zero more frequently exhibited elevated levels of IL-1β and NLRP3 than children with severity score zero. This suggests a possible involvement of these genes in hMPV infected children with severe disease.

As mentioned earlier, previous studies on RSV have shown a variation in disease severity for different genotypes, but similar studies on hMPV have been inconclusive [5–8]. Consequently, we wanted to see if our data suggested any differences in gene expression or disease severity for the hMPV A2 and B2 genotypes. However, we did not detect any differences between A2 and B2 with respect to expression profiles except for IFN-β and TNF-α which was significantly higher expressed in A2 and B2 positive patients, respectively, at mRNA level. Further, we did not detect any differences in the length of hospital stay or disease severity in the A2 and B2 infected children.

Our study population includes infants and up to 8 years old children. Consequently there is a risk that children with both primary and secondary hMPV infections are included. These might experience different immune responses. To address this we compared children less than one year of age with older children. We found no differences in gene expression profiles (data not shown). In addition, the average age of children with severity score 1–4 was not significantly different from those with severity score zero (P = 0.28, Student’s t-test), indicating that the youngest children where not more disposed to severe disease compared to the older. Of note, there was a difference in age distribution between hMPV-infected and control children. The controls were symptom-free and screened for the same airway pathogens as the patient samples, and no pathogens were detected. Hence, we would not anticipate that the inflammatory genes are expressed above a basal level in the controls. Further, to our knowledge, no studies so far have shown that basal expression is significantly different in children less than one year and older.

Our use of clinical samples from selected, well-classified patients is a strength of the study. For studies aimed to examine immune responses to a particular viral infection in vivo it is a challenge, especially in children, to obtain sufficient number of samples collected at similar conditions. In particular, interfering viral and bacterial co-infections are common. In our study population extensive viral analyses of each sample revealed that nearly half of the population had viral co-infections and were excluded from further analysis. In addition, bacterial infections were unlikely because most children had received one or more conjugated pneumococcal vaccinations, and only patients with maximal CRP levels less than 100 mg/L were included. Furthermore, we only included samples that were collected within the first week after onset of symptoms. Finally, separating the samples based on the two most frequent hMPV genotypes enabled us to see if there was a genotype-specific cytokine gene expression profile or differences in disease severity. This allowed us to study the initial host immune response towards particular hMPV genotypes, which extends current literature. However, our selection criteria limited the total number of patients that were eligible for our study. Consequently, it will be necessary to confirm our findings in other cohorts, in particular with respect to inflammasome-associated genes in children with severe RTI.

In summary, our study has identified inflammasome components that may be key players in the innate immune response against hMPV in children. The recent discovery of NLRP3 inhibitors [41] that may prevent or even treat RTI-mediated inflammation, emphasizes the need to establish the role of inflammasomes in the response to RTI in children. Our study provide an important contribution in this regard.

## Supporting Information

S1 Table Clinical evaluation of the hMPV infected patients. (DOCX)

S2 Table qPCR primers included in the study. (DOCX)
