# Supplementary material for: Cytokine Profiles in Human Metapneumovirus Infected Children: Identification of Genes Involved in the Antiviral Response and Pathogenesis
Source: PLoS One. 2016 May 12;11(5):e0155484. doi: 10.1371/journal.pone.0155484 (PMC4865088; doi:10.1371/journal.pone.0155484)
Supplement: S1 Table — (DOCX) [file pone.0155484.s001.docx]

**S1 Table: Clinical evaluation of the hMPV infected patients.**

**A) A2 positive patients.**

| Patient | Age,  months | Severity score | Hosp., days | CRP, mg/L | hMPV Ct | GT | Diagnosis | Comorbidities | Steroids |
| --- | --- | --- | --- | --- | --- | --- | --- | --- | --- |
| 1 | 29.4 | 0 | 1 | 30 | 19.7 | A2a | RP, fever cramps | None | No |
| 2 | 5.1 | 0 | 1 | n.a. | 21.8 | A2a | Rhinitis, bronchiolitis | Laryngomalacia | No |
| 3 | 83.0 | 0 | 1 | <5 | 24.9 | A2b | LRTI, fever cramps | None | No |
| 4 | 11.4 | 4 | 16 | 44 | 20.4 | A2b | Asthma exacerbations | Asthma | Yes |
| 5 | 18.5 | 1 | 3 | 51 | 19.2 | A2a | Bronchiolitis, tonsilitis | None | Yes |
| 6 | 43.6 | 0 | 1 | 17 | 21.0 | A2b | Asthma exacerbation, RP | Asthma | Yes |
| 7 | 14.7 | 0 | 3 | 29 | 23.2 | A2a | Bronchiolitis | None | No |
| 8 | 4.6 | 0 | 1 | <5 | 22.1 | A2a | Bronchiolitis | None | No |
| 9 | 22.0 | 0 | 2 | 7 | 21.6 | A2a | Bronchiolitis, RP | None | No |
| 10 | 9.6 | 1 | 4 | 95 | 25.8 | A2b | Pneumonia, RP, tonsilitis | None | No |
| 11 | 11.5 | 2 | 5 | 80 | 24.5 | A2b | Pneumonia, RP, SO | None | No |
| 12 | 29.9 | 2 | 6 | 40 | 23.3 | A2b | Asthma exacerbation | PTB, asthma | Yes |
| 13 | 0.7 | 3 | 6 | 44 | 26.2 | A2b | Bronchiolitis, RP | None | No |
| 14 | 24.9 | 2 | 10 | 84 | 28.8 | A2b | Pneumonia, RP, SO | None | No |
| 15 | 29.7 | 0 | 1 | 33 | 27.3 | A2a | Bronchiolitis, RP, PO | None | Yes |

**B) B2 positive patients.**

| Patient | Age,  months | Severity score | Hosp., days | CRP, mg/L | hMPV, Ct | GT | Diagnosis | Comorbidities | Steroids |
| --- | --- | --- | --- | --- | --- | --- | --- | --- | --- |
| 1 | 1.9 | 3 | 8 | 25 | 16.5 | B2 | Bronchiolitis | PTB | No |
| 2 | 22.4 | 4 | 6 | 74 | 27.4 | B2 | Pneumonia, RP | PTB, CP, PH,  TBM, BPD, asthma | Yes |
| 3 | 3.1 | 1 | 4 | 5 | 21.7 | B2 | Bronchiolitis, rhinitis | PTB | No |
| 4 | 17.5 | 1 | 3 | <5 | 20.6 | B2 | Asthma exacerbation | PTB, asthma | Yes |
| 5 | 3.1 | 0 | 3 | 10 | 23.7 | B2 | Bronchiolitis | None | No |
| 6 | 1.0 | 0 | 3 | 39 | 33.1 | B2 | Bronchiolitis | None | No |
| 7 | 91.9 | 0 | 1 | 50 | 20.8 | B2 | Pneumonia | PTB, pollen allergy | No |
| 8 | 56.6 | 1 | 2 | 16 | 30.8 | B2 | Asthma exacerbation, SO | Asthma, DS | Yes |
| 9 | 33.9 | 0 | 1 | 5 | 24.6 | B2 | Bronchiolitis, URTI | PTB, BPD, CP, asthma | Yes |
| 10 | 11.8 | 0 | 1 | <5 | 29.1 | B2 | Pneumonia, RP, SO | Eczema | No |
| 11 | 7.7 | 2 | 4 | 42 | 30.5 | B2 | Bronchiolitis, RP, SO | CPAP inhalation | Yes |
| 12 | 1.6 | 0 | 1 | <5 | 31.5 | B2 | Bronchiolitis, RP | None | No |
| 13 | 31.7 | 0 | 4 | 13 | 27.3 | B2 | Rhinopharyngitis, SO | Isovaleric acidemia | No |
| 14 | 33.9 | 1 | 2 | 57 | 33.9 | B2 | LRTI, tonsilitis, serous otitis | None | No |
| 15 | 17.5 | 1 | 4 | 47 | 30.2 | B2 | Bronchiolitis, purulent otitis | None | No |

**C) Controls.**

| Control | Age,  months | Virus PCR/culture | Chronic diseases | Steroids |
| --- | --- | --- | --- | --- |
| 1 | 42.3 | Negative | No | No |
| 2 | 26.2 | Negative | No | No |
| 3 | 26.5 | Negative | Eczema | No |
| 4 | 10.9 | Negative | No | No |
| 5 | 46.6 | Negative | No | No |
| 6 | 10.4 | Negative | No | No |
| 7 | 46.5 | Negative | No | No |
| 8 | 47.3 | Negative | No | No |
| 9 | 3.9 | Negative | No | No |
| 10 | 57.4 | Negative | No | No |

Abbreviations: BPD, bronchopulmoary dysplasia; CP, cerebral palsy; CPAP, continuous positive airway pressure; CRP, c-reactive protein; Ct, cycle threshold; DS, Downs syndrome; GT, hMPV genotype; Hosp., hospitalization; hMPV, human metapneumovirus; N.a., not available; PH, pulmonary hypertension; PTB, pre-term birth; RP, rhinopharyngitis; SO, simplex otitis; TBM, tracheobronchomalasia; U/L-RTI, unspecified upper/lower respiratory tract infection.
