# Supplementary material for: Cytokine Profiles in Human Metapneumovirus Infected Children: Identification of Genes Involved in the Antiviral Response and Pathogenesis
Source: PLoS One. 2016 May 12;11(5):e0155484. doi: 10.1371/journal.pone.0155484 (PMC4865088; doi:10.1371/journal.pone.0155484)
Supplement: S2 Table — (DOCX) [file pone.0155484.s002.docx]

**S2 Table: qPCR primers included in the study.**

| Target gene | Forward sequence (5' - 3') | Reverse sequence (5' - 3') |
| --- | --- | --- |
| GAPDH | GAAGGTGAAGGTCGGAGTC | GAAGATGGTGATGGGATTTC |
| IFN-β | GCCGCATTGACCATCTATGAGA | GAGATCTTCAGTTTCGGAGGTAAC |
| IFN-γ | GGCATTTTGAAGAATTGGAAAG | TTTGGATGCTCTGGTCATCTT |
| IL-28 (IFN-λ2/3) | AGGGCCAAAGATGCCTTAG | CAGCTCAGCCTCCAAAGC |
| IL-1β | TACCTGTCCTGCGTGTTGAA | TCTTTGGGTAATTTTTGGGATCT |
| IL-18 | TTGCTGAGCCCTTTGCTC | GCTTTAGCAGCCAGAGTTGG |
| NLRP3 | TGATGTTCTGTGAAGTGCTGAA | CGCACTTTTTGTCTCATAATTGA |
| IP-10 | TCGAAGGCCATCAAGAATTT | GCTCCCCTCTGGTTTTAAG |
| TNF-α | CAGCCTCTTCTCCTTCCTGAT | GCCAGAGGGCTGATTAGAGA |
| IL-6 | GATGAGTACAAAAGTCCTGATCCA | CTGCAGCCACTGGTTCTGT |
| ISG54 (IFIT2) | TGGTGGCAGAAGAGGAAGAT | GTAGGCTGCTCTCCAAGGAA |
| IκBα | GTCAAGGAGCTGCAGGAGAT | ATGGCCAAGTGCAGGAAC |
